# Health system resilience: quantifying the dynamic impact of environmental shocks on health service utilization using an interrupted time series and time-series forecasting approach in Western Province, Zambia

**DOI:** 10.1016/j.joclim.2026.100672

**Published:** 2026-04-10

**Authors:** Cameron B. Chiarot, Craig R. Janes, Fastone Goma, Karen A. Grépin, Abel Torres-Espin, Elizabeth J. Mroz, Mark W. Smith, Zahid A. Butt

**Affiliations:** a School of Public Health Sciences, Faculty of Health, University of Waterloo, Waterloo, Ontario, Canada; b Centre for Primary Care Research, Kasama Road, Lusaka, Zambia; c Li Ka Shing Faculty of Medicine, School of Public Health, The University of Hong Kong, Pokfulam, Hong Kong Special Administrative Region, China; d Department of Neurological Surgery, University of California San Francisco, San Francisco, CA, USA; e School of Geography and Water@Leeds, University of Leeds, Leeds, United Kingdom

**Keywords:** Health system resilience, Time-Series Forecasting, Interrupted Time Series, Climate Shocks, Health Service Utilization, Zambia

## Abstract

**Introduction:**

In low- and lower-middle-income nations, advancements toward Universal Health Coverage (UHC) are progressively jeopardized by the combined effects of acute shocks, such as floods and pandemics, alongside chronic health system stressors. This study employs a multi-model time-series methodology to dynamically forecast health service utilization and assess the impacts of major shocks in Western Province, Zambia.

**Materials and methods:**

We conducted a longitudinal ecological study utilizing 73 months of routine health data from 62 healthcare facilities (October 2017 to September 2023). We used Prophet and Hierarchical Time Series (HTS) models for forecasting and an Interrupted Time Series (ITS) analysis to quantify the impacts of a drought, the COVID-19 pandemic, and a double-peak flood event.

**Results:**

The Prophet model was the most accurate forecasting tool (MAPE = 7.22 %). The ITS analysis demonstrated that each shock distinctly affected health service utilization. The drought was associated with an immediate decline in utilization (level change = −0.105, *p* < 0.001), while the COVID-19 pandemic resulted in an immediate increase (level change = 0.069, *p* < 0.001). The 2023 flood event showed no immediate impact but was associated with a significant positive trend change (trend change = 0.019, *p* < 0.001).

**Discussion:**

This study provides innovative empirical evidence demonstrating that the interaction between environmental shocks and health system stressors dynamically affects health service utilization.

**Conclusion:**

The findings underscore the significance of adopting multi-model predictive strategies to produce early warnings and to inform targeted, data-driven interventions to strengthen health system resilience.

## 1 Introduction

The diverse health effects of climate change threaten the achievement of Universal Health Coverage (UHC) and the stability of health systems in vulnerable regions [1,2]. Health system resilience—the ability to anticipate, absorb, and adapt to shocks—is a vital component of climate adaptation [3,4]. In low- and lower-middle-income countries (LMICs), health systems often face acute shocks like floods and pandemics that converge with chronic stressors such as poor infrastructure and barriers to care [5,6]. These shocks rarely occur in isolation, instead interacting with systemic vulnerabilities to disrupt equitable access to health services [7]. While conceptual frameworks like syndemic theory offer valuable insights, a gap persists in quantifying the dynamic, synergistic effects between environmental shocks and health system stressors over time [8].

Effectively addressing this challenge requires a multi-model strategy. While traditional methods like ARIMA often struggle to manage the non-linear trends and complex seasonality found in health data from fragile settings, this study employs the more robust Prophet model [9,10]. Accordingly, this study employs the Prophet model as a more robust and contemporary alternative. Building on the Prophet forecasts, this study also incorporates a Hierarchical Time Series (HTS) model to analyze data across different administrative levels and an Interrupted Time Series (ITS) analysis to assess the inferential effects of specific shocks [11,12].

While equitable access is a core UHC principle, it is challenging to measure comprehensively across space and time. Therefore, this study uses general utilization (i.e., monthly outpatient visits) as a practical proxy for *realized access* [13].This metric is relevant in LMICs, where primary health facilities are the initial point of contact for most of the population. Analyzing this measure offers insights into the resilience of the health system's frontline, where the impacts of shocks are most acutely felt and interventions are often implemented.

Western Province (WP), Zambia, is an exemplary setting to investigate these converging challenges. The region is characterized by the vast Barotse Floodplain, where significant seasonal inundation—while vital to the local ecology and economy—constitutes a recurrent environmental shock. This shock intersects with chronic health system stressors, including low facility density and substantial geographic barriers to care [14,15]. To examine these interactions, this study employs the novel econosyndemic framework.^1^ An extension of syndemic theory, this framework models the dynamic interplay between macro-level shocks and micro-level health system stressors on health service utilization over time. The specifics of this framework are detailed by Singer [16].

This study has two main objectives. First, we apply and evaluate advanced time-series methods, including Prophet and Hierarchical Time Series (HTS) models, to forecast health service utilization and to assess whether an exogenous flood variable improves forecast precision. Second, we use an Interrupted Time Series (ITS) analysis to evaluate the immediate and long-term impacts of three distinct shocks: the 2019 drought, the 2021 COVID-19 pandemic, and the 2023 double-peak flood. The results provide a comprehensive and dynamic understanding of health service utilization within a climate-vulnerable context, offering valuable insights for planning, policy formulation, and health system enhancement.

## 2 Materials and methods

### 2.1 Study design and setting

This study employed a longitudinal ecological study design, using time-series analysis to examine patterns and determinants of health service utilization. This approach facilitated the assessment of temporal trends and the impacts of specific shocks on health outcomes within an existing population without the need for direct intervention. The three macro-level shocks analyzed included a drought in 2019, the COVID-19 pandemic (i.e., SARS-CoV-2), and a complex flooding event in 2023. The analysis focused on a longitudinal dataset comprising facility-month observations, with health facilities serving as the primary units of analysis. The research was conducted in Western Province (WP), Zambia, a region in Sub-Saharan Africa with an estimated population of 1.36 million in 2022 [17]. Western Province is notably characterized by the Barotse Floodplain, an extensive wetland system along the Zambezi River, distinguished by seasonal flooding variations [14]. Healthcare services are provided through a network of first-level health facilities (i.e., primary health care), including health centers and health posts [18]. The study period spanned from October 2017 to September 2023, covering a comprehensive temporal window of 73 months for analysis. The final analytic dataset comprised 62 public health centres and health posts across seven administrative districts within WP, where monthly flood exposure data were available.

### 2.2 Data sources and variables

Longitudinal data on general utilization (i.e., outpatient visits) were obtained from Zambia's Health Management Information System (HMIS), its national routine health information system [18,19]. Supplementary data for population denominators and geographic access modelling were derived from the Spatially Defined Catchment Area (SCSO) and Population Under Rooftop (PUR) methodology,^2^ which integrates official Central Statistical Office (CSO) population estimates with high-resolution, satellite-derived gridded population data from Meta's High Resolution Settlement Layers (HRSL) [20,21]. Flood exposure data were obtained from the LISFLOOD-FP hydrodynamic inundation model, a widely used model that simulates river hydrodynamics to predict flood extent and depth, which provides average monthly outputs of floodwater dynamics, including water depth with a spatial resolution of 900m [14,22]. It is important to note that the flood exposure data represents a single, district-wide monthly average for the region. As such, the variable captures the overall temporal dimension of seasonal flooding but does not account for finer-scale, localized variations in flood intensity across different districts or facility catchment areas.

The primary dependent variable, general utilization (i.e., outpatient visits), was selected as a proxy for realized access and defined as the monthly proportion of health facility visits. Alternative metrics, such as ambulance callouts or emergency admissions, were excluded as they are negligible in this setting due to the lack of formal emergency transport infrastructure in the remote floodplain districts. The monthly proportion of outpatient visits was selected as the primary outcome to normalize utilization across health facilities with vastly different catchment population sizes and to account for population growth over the 73-month study period. This approach ensures that trends in utilization are not confounded by underlying demographic changes and maintains methodological consistency with related analyses in previous research. This proportion was calculated by dividing the monthly number of reported outpatient visits by the estimated general population within each health facility's catchment area. To ensure compatibility with the Beta regression models used in the ITS analysis, a continuity transformation was applied to the entire vector of observed proportions to constrain the data to the open interval (0,1). This adjustment, based on the Smithson and Verkuilen approach, modified the proportion as follows: $y^{\prime }=y\cdot \left(n-1\right)+0.5/n$ where $y^{\prime }$ is the transformed proportion used as the outcome variable in the model, constrained to an open interval (0,1), y is the original proportion of general utilization (i.e., outpatient visits), and n is the total number of observations used for the transformation [23].

Several independent variables and covariates were constructed to account for geographic, environmental, and temporal factors. The characteristics of each health facility, including its unique name, geographic coordinates (latitude and longitude), type (Health Centre or Health Post), and location classification (rural or urban), were used. The administrative district of each health facility was included as a categorical variable, with *Kalabo* district serving as the *reference* category among the seven study districts. Flood depth, derived from the LISFLOOD-FP hydrodynamic model, was measured in meters [22]. This continuous variable was also categorized into three levels: *Low* (<0.2 m; *reference*), Medium (0.2 − 0.4 m), and High (>0.4 m). The reference category for flood depth was established as *Low* (<0.2 m). Within the context of the Barotse Floodplain, absolute *zero* water levels are geographically indistinct from low levels owing to the wetland environment. A depth of <0.2 m (approximately 8 inches) was selected as the reference point because it remains accessible by foot, whereas depths exceeding this threshold generally require canoe transportation, consequently affecting access dynamics. A one-month lag version of the flood depth variable was incorporated into the exogenous variable Prophet models and the final ITS model to capture the delayed effects of flooding. Population denominators were adjusted using district-specific annual Population Growth Rates (PGRs) derived from 2010–2020 Zambia Statistics Agency estimates (Kalabo: 2.5 %; Limulunga: 1.6 %; Lukulu: 4.2 %; Mitete: 3.1 %; Mongu: 3.6 %; Nalolo: 2.4 %; Sikongo: 2.3 %) to account for dynamic changes in catchment area populations over time [17]. Geographic access was quantified using estimated travel time, which was calculated as the straight-line distance between the population-weighted centroid of each health facility's catchment area (PUR centroid) and the health facility. The *law of cosines* was applied to calculate this distance defined by: $d_{ij}=R\cdot \arccos\left(\sin\left(\phi_{i}\right)\sin\left(\phi_{j}\right)+\cos\left(\phi_{i}\right)\cos\left(\phi_{j}\right)\cos\left(\lambda_{j}-\lambda_{i}\right)\right)$ where $d_{ij}$ is the straight-line distance between point i (PUR centroid) and point j (health facility), ϕ is the latitude (radians), λ is the longitude (radians), and R is the Earth’s radius ≈ 6371 km. This distance was then divided by an assumed walking speed of 5 km/hr to estimate travel time in minutes, which was subsequently categorized into three levels: 0 − 2 hrs (*reference*), 2 − 5 hrs, and >5 hrs. To account for underlying temporal trends and seasonal patterns, the models included a linear time trend representing the number of months since the study start and two harmonic terms, which were calculated as the sine and cosine of the month number over a 12-month cycle. The harmonic time terms were given by: $time_{sin}=\text{sin}\left(2\pi t/12\right)$ and $time_{cos}=\text{cos}\left(2\pi t/12\right)$.

### 2.3 Data preparation and cleaning

The analytic dataset was refined to include exclusively data from the designated study period, spanning October 2017 to September 2023, and to encompass a subset of 62 health facilities distributed across the seven administrative districts where complete monthly flood exposure data was obtainable. This refinement process yielded a final dataset comprising 4,254 facility-month observations for analytical purposes. The dataset was subsequently divided into two subsets to facilitate the primary analytical components of the study: time-series forecasting and Interrupted Time Series (ITS) analysis. This approach ensured that the specific data formatting requirements of each modelling approach were satisfied without compromising the integrity of the original dataset. All facility-month observations were aggregated to generate a single monthly time series for provincial-level forecasting. As the outcome variable represented a proportion, a weighted aggregation method was employed to ensure an accurate depiction of utilization at the provincial level. First, the monthly visit count for each facility was recalculated by multiplying its utilization proportion by its corresponding population denominator. These counts were then summed across all facilities for each month. Finally, a single, aggregate provincial utilization proportion was calculated by dividing the total monthly visits by the total provincial population for that month. This approach avoids potential bias that could arise from simply averaging the individual facility proportions. District-level data were aggregated similarly for the disaggregated forecasting models. A comprehensive analysis was conducted, and observations were excluded if the primary outcome or key predictors were missing. Without a time-series imputation method, this approach ensured that all models were fitted to a complete dataset, which is essential for the stability and interpretability of regression models. The missingness rate for the primary outcome, general utilization, was approximately 5 % of facility-month observations. The application of lagged flood variables in certain analyses also necessitated the systematic exclusion of the first and last months of data for each facility.

### 2.4 Statistical analysis

**Model Selection Rationale:** This study employed a dual-modelling strategy to address two distinct analytical objectives: forecasting accuracy and causal inference. Firstly, to establish an early warning system for health service utilization, the Prophet model was selected. Prophet is an additive regression model optimized for predictive accuracy, minimizing RMSE (Root Mean Square Error) and MAPE (Mean Absolute Percentage Error), in the presence of non-linear trends and complex seasonality typical of routine health data. Secondly, to quantitatively assess the immediate and long-term impacts of environmental shocks, a Generalized Linear Mixed Model (GLMM) was utilized within an Interrupted Time Series (ITS) framework. Unlike Prophet, the GLMM framework facilitates rigorous testing of causal hypotheses about level and slope changes, while explicitly modelling the data’s Beta distribution and accounting for hierarchical clustering.

**Time series forecasting:** A comparative analysis was performed to identify the most accurate forecasting model for general utilization. Four models were evaluated: an autoregressive integrated moving average (ARIMA), a seasonal ARIMA (SARIMA), an exponential smoothing model (ETS), and an additive model that accounts for non-linear trends and seasonality (Prophet). The Prophet model is based on a decomposable time series model that captures a trend, seasonality, and holidays and is given by $y\left(t\right)=g\left(t\right)+s\left(t\right)+h\left(t\right)+\epsilon_{t}$ where y(t) is the forecasted proportion of general utilization at time t, g(t) is the non-linear trend function, s(t) is the seasonal component, h(t) is the holiday effect component (excluded in this model), and $\epsilon_{t}$ is the error term. The Prophet model captures seasonality automatically using a Fourier series, so external harmonic terms were not specified for the forecasting component of the analysis. The models were trained on data from October 2017 to December 2022 and validated on a 9-month test set from January 2023 to September 2023. Forecast accuracy was assessed using three standard metrics: Root Mean Squared Error (RMSE), Mean Absolute Error (MAE), and Mean Absolute Percentage Error (MAPE). The model with the lowest MAPE was selected for further analysis. The results of this comparison are presented in Table A.1 and visualized in Figure A.1. The Prophet model was implemented using the default hyperparameters of the *prophet* package in R to ensure reproducibility [24].

**Fig. 1:**
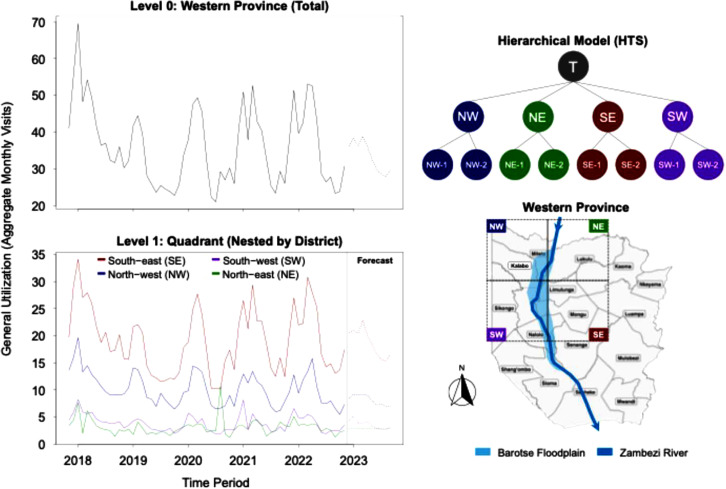
Hierarchical Time Series (HTS) forecasts of general utilization extending to December 2024. The model aggregates predictions from 62 health facilities (Level 2), which are nested within the four geographic quadrants shown on the map (Level 1).

**Hierarchical time series:** To forecast general utilization across different levels of aggregation, a hierarchical time series (HTS) model was developed [25]. The hierarchy was defined with three levels: Level 0 represented the total for WP, Level 1 comprised four geographic quadrants, and Level 2 comprised the 62 individual health facilities. The base time series was a matrix of monthly visit counts for each facility. This hierarchy can be described mathematically as: $y_{t}=S\beta_{t}$ where $y_{t}$ is a vector of the total general utilization visits at each level of the hierarchy at time t, S is the summation matrix that maps the bottom-level series to their corresponding aggregates at high levels, and $\beta_{t}$ is a vector of the bottom-level series (i.e., individual health facility general utilization visits) at time t. The forecast reconciliation was performed using a bottom-up (*bu*) approach, where individual facility forecasts were aggregated to generate forecasts for the higher levels of the hierarchy (Fig. 1).

**Exogenous variable modelling:** To assess the impact of environmental shocks on forecast accuracy, two augmented Prophet models were developed at the district level. These models incorporated flood data as an exogenous regressor. The Prophet model with an exogenous regressor is extended by adding a linear term for the new variable, such that the model becomes: $y\left(t\right)=g\left(t\right)+s\left(t\right)+h\left(t\right)+\beta X_{t}+\epsilon_{t}$ where y(t) is the forecasted proportion of general utilization at time t, g(t) is the non-linear trend function, s(t) is the seasonal component, h(t) is the holiday effect component (excluded in this model), β is the coefficient for the exogenous regressor, $X_{t}$ is the value of the exogenous regressor (i.e., continuous floodwater depth measured in meters or a categorical dummy variable) at time t, and $\epsilon_{t}$ is the error term. The first model included a continuous flood depth variable, while the second used a categorical flood level variable (low, medium, and high). The forecast accuracy of these flood-augmented models was compared to a baseline Prophet model for each of the seven districts. The performance of the models, as measured by RMSE, MAE, and MAPE, is detailed in Table A.4 and visualized for key districts in Figure A.3.

**Interrupted time series:** The immediate (level) and long-term (trend) impacts of three major interruptions on health utilization were evaluated using an interrupted time series (ITS) design [26]. A generalized linear mixed-effects model (*glmmTMB*) was employed, which is well-suited for longitudinal data with a hierarchical structure [27]. The outcome, general utilization, was assumed to follow a Beta distribution with a logit link function. The fixed effects in the model included a linear time trend (months since study start), and, to explicitly control for seasonal patterns, harmonic terms for seasonality (sine and cosine), and fixed effects for each of the seven administrative districts, with *Kalabo* district as the *reference*. The model also included a random intercept for each health facility to account for unobserved facility-level heterogeneity. The three primary interruptions were specified as changepoints in the time series and were modelled with both level and trend variables given by:

$$L_{m}=\left\{ \begin{array}{c} 0,\;\text{if}\;t<C_{m}\\ 1,\;\text{if}\;t\geq C_{m} \end{array}\right.\;\text{and}\;T_{m}=\left\{ \begin{array}{c} 0,\;\text{if}\;t<C_{m}\\ t-C_{m},\;\text{if}\;t\geq C_{m} \end{array}\right.$$

where $L_{m}$ is the level variable for interruption m, $T_{m}$ is the trend variable for interruption m, t is time measured as months since start, and $C_{m}$ is the changepoint date (in months) for interruption m. The model's final structure is defined by:

$$\begin{array}{c} logit\left(\mu_{it}\right)=\beta_{0}+\beta_{1}\left(t\right)+\beta_{2}\left(\sin\left(t\right)\right)+\beta_{3}\left(\cos\left(t\right)\right)+\\ \sum_{j=1}^{k}\beta_{j+3}\left(District_{j}\right)+\sum_{m=1}^{3}\left[\delta_{m}\left(L_{m}\right)+\gamma_{m}\left(T_{m}\right)\right]+u_{i} \end{array}$$

where $logit(\mu_{it})$ is the log-odds of the mean general utilization proportion for facility i at time t, $\beta_{0}$ is the intercept, $\beta_{1}\left(t\right)$ is the linear time trend, where t is months since start, $\beta_{2}\left(\sin(t)\right)+\beta_{3}\left(\cos(t)\right)$ are the harmonic terms used for seasonality, $\sum_{j=1}^{k}\beta_{j+3}\left(District_{j}\right)$ is the fixed-effects for the k districts, $\delta_{m}\left(L_{m}\right)$ is the immediate change in the level of general utilization for interruption m, $\gamma_{m}\left(T_{m}\right)$ is the change in the trend of general utilization for interruption m, and $u_{i}$ is the random intercept for each health facility i. To visualize the impact of each interruption, counterfactual time series were generated by setting the relevant level and trend variables to zero for all post-intervention time points in the final model's linear predictor [26]. This approach facilitated a comparative analysis between the predicted outcomes of the factual model and a hypothetical scenario wherein a specific interruption had not occurred. The visualization of these scenarios is presented in Fig. 2b The results of the final model, including fixed-effects estimates, 95 % confidence intervals, standard errors, and *p*-values, are summarized in Table 2. This table provides the statistical evidence for the immediate and long-term effects of each interruption on overall utilization. The entire time series of general utilization and flood depth is depicted in Fig. 2a to offer an environmental context for the ITS model.

**Fig. 2a:**
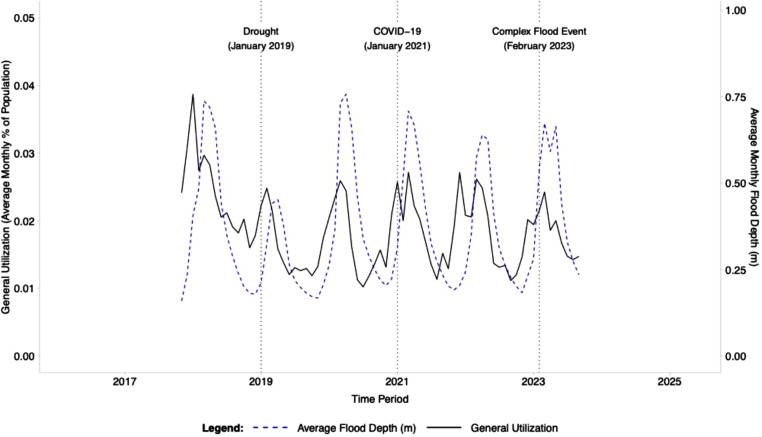
Time series of general utilization and flood depth. The observed monthly time series of general utilization and flood depth spans from October 2017 to September 2023. The solid black line represents the monthly average utilization (% of population, left axis), while the dashed blue line illustrates the average monthly flood depth (m, right axis). This plot provides environmental context for the three major interruptions—Drought (January 2019), COVID-19 (January 2021), and the Complex Flood Event (i.e., February 2023 double-peak flood)—which are analyzed in the subsequent ITS model (Fig. 2b).

### 2.5 Statistical inference

For the comparative time-series models, forecast accuracy was evaluated using RMSE, MAE, and MAPE. The model with the lowest MAPE was selected as the best-performing model. The forecast utilization flag thresholds presented in Table A.2 were defined based on the quartiles of the historical distribution of monthly general utilization from 2017–2023. For the ITS analysis, the Beta regression model was based on a two-sided significance level of α=0.05. For each predictor, final model outputs include the logit-scale coefficient, standard error (SE), adjusted odds ratio (AOR), and the corresponding 95 % confidence interval (CI) and *p*-value. An association was considered statistically significant if the *p*-value was less than 0.05 and the 95 % CI for the AORs did not include 1.0.

### 2.6 Model diagnostics

The assessment of model assumptions was carried out through the analysis of simulation-based residuals. The distribution of these residuals was meticulously examined to evaluate their uniformity and heteroscedasticity. Furthermore, the variance inflation factor (VIF) was determined using the *performance* package to identify potential multicollinearity among the fixed effects of the model. Notably, a diagnostic evaluation of the residuals revealed significant autocorrelation, which could not be rectified due to convergence failures encountered when an autoregressive component was incorporated. Consequently, *p*-values should be interpreted with caution, as autocorrelation can lead to an underestimation of standard errors, potentially making some results appear more statistically significant than they truly are. Therefore, the findings should be viewed as strong evidence of association rather than precise causal estimates. All data processing, visualization, and statistical analyses were performed utilizing R statistical software (version 4.3.1) [28,29].

### 2.7 Ethics approval

Ethical approval for this study was obtained from the University of Waterloo Human Research Ethics Board (ORE-43,883), the University of Zambia Biomedical Research Ethics Committee (Ref. No. 2472–2021; renewed February 9, 2024), the National Health Research Authority in Zambia (Ref. No. NHRA000009/10/07/2022), and the Provincial Ministry of Health, WP. Permission was granted for the secondary analysis of a large, de-identified administrative dataset. Patients or members of the public were not directly involved in designing, conducting, or reporting this research.

## 3 Results

### 3.1 Descriptive statistics

The final analytic dataset consisted of 4,254 facility-month observations, derived from a network of 62 primary care facilities across seven administrative districts in WP. The average monthly proportion of general utilization over the entire study period was 0.000189 (SD = 0.000126), with a median of 0.000179. Geographic access, measured by the average travel time to a health facility (based on straight-line distance and an assumed walking speed of 5km/hr), was 63.3 min (SD = 42.0). Most observations originated from rural health facilities, representing 93 % of the total, with health centers and health posts comprising 52 % and 48 % of the data, respectively. Over the entire 73-month study period, the distribution of monthly flood severity categories was 14 % for low flood exposure, 49 % for medium, and 37 % for high. A comprehensive summary of these descriptive statistics, stratified by each of the seven districts, is provided in Table 1.

**Table 1:** Descriptive statistics of key variables stratified by district.

Variable	Overall	Kalabo	Limulunga	Lukulu	Mitete	Mongu	Nalolo	Sikongo
Observations (N)	4,254	948	1,335	207	125	1,083	486	701
**Outcome**								
General Utilization								
Proportion	0.000189	0.000192	0.000197	0.000244	0.000192	0.000178	0.000159	0.000168
Std. Dev.	(0.000126)	(0.000127)	(0.000136)	(0.000164)	(0.000106)	(0.000117)	(0.000087)	(0.000075)
**Predictors**								
Health Facilities								
Type								
Health Centre	2,226 (52 %)	406 (43 %)	704 (53 %)	69 (33 %)	0 (0 %)	701 (65 %)	276 (57 %)	70 (100 %)
Health Post	2,028 (48 %)	542 (57 %)	631 (47 %)	138 (67 %)	125 (100 %)	382 (35 %)	210 (43 %)	0 (0 %)
Location								
Rural	3,975 (93 %)	948 (100 %)	1,335 (100 %)	207 (100 %)	125 (100 %)	804 (74 %)	486 (100 %)	70 (100 %)
Urban	279 (7 %)	0 (0 %)	0 (0 %)	0 (0 %)	0 (0 %)	279 (26 %)	0 (0 %)	0 (0 %)
Ave. Travel Time (mins)	63.3 (42.0)	83.3 (51.7)	57.5 (34.8)	95.0 (19.3)	79.4 (9.5)	44.3 (37.0)	66.8 (37.9)	48.79 (0.0)
Flooding								
Water Depth (m)	0.37 (0.18)	0.37 (0.18)	0.37 (0.18)	0.37 (0.18)	0.37 (0.18)	0.37 (0.18)	0.37 (0.18)	0.37 (0.18)
Flood Severity (lag)								
Low	598 (14 %)	139 (15 %)	186 (14 %)	29 (14 %)	16 (13 %)	149 (14 %)	69 (14 %)	10 (14 %)
Medium	2,095 (49 %)	462 (49 %)	657 (49 %)	104 (50 %)	63 (50 %)	534 (49 %)	241 (50 %)	34 (49 %)
High	1,561 (37 %)	347 (37 %)	492 (37 %)	74 (36 %)	46 (37 %)	400 (37 %)	176 (36 %)	26 (37 %)

**Note:** Data are presented as *n*(%) for categorical variables and mean (standard deviation) for continuous variables. Statistics were calculated from 4,254 facility-month observations from 62 unique health facilities between October 2017 and September 2023. "Flood Severity (lag)" was based on the flood depth from the preceding month.

### 3.2 Time-series forecasting

The time-series forecasting analysis was conducted in three stages: a model benchmarking exercise, a hierarchical forecast, and an assessment of exogenous variables. A comparative analysis of four time-series models revealed that the Prophet model provided the most accurate forecasts for general utilization. As detailed in Tables A.2 and A.4, the Prophet model achieved the lowest MAPE at 7.22 % on the 9-month test set, which is an *excellent* forecast based on the pre-defined interpretation criteria. A hyperparameter grid search was conducted to validate the Prophet model specification; however, default parameters yielded superior accuracy (MAPE = 7.22 %) compared to tuned alternatives (MAPE = 16.40 %), supporting the robustness of the baseline model. In comparison, the Exponential Smoothing (ETS) model performed well with an MAPE of 13.56 %, while the ARIMA and SARIMA models both yielded MAPE values of 16.30 %. This superior performance of the Prophet model led to its selection for all subsequent forecasting analyses. A visual representation of the Prophet model's aggregate forecast for the entire province is presented in Figure A.1, which displays the model’s fit on the training data and its forecast on the test set, including a 95 % confidence interval.

The Prophet model was employed to forecast overall utilization for the period from September 2023 to September 2024, with the results delineated at the district and quarterly levels in Table A.2. A summary of the metrics and thresholds utilized for this analysis is provided in Table A.3. The forecasts underscored notable variations in anticipated utilization across districts and quarters. For instance, several districts, including Kalabo and Limulunga, are projected to experience periods of over-utilization in Q1 2024, with forecasted percentages significantly exceeding the 0.023 % threshold (Table A.4). Conversely, districts such as Sikongo and Lukulu are consistently projected to fall within the severe under-utilization category for most of the forecasted period, exhibiting substantial negative deviations from their baseline values (Tables A.2 and A.3).

The hierarchical time series (HTS) model offered a coherent and reconciled forecast of provincial utilization by consolidating predictions derived from individual health facilities. This analysis validated an overarching downward trend in utilization across most regions of the province, accompanied by notable seasonal variations. The reconciled top-level forecast for the entire province achieved a Mean Absolute Percentage Error (MAPE) of 14.5 % on the 9-month test set. The forecasts for WP (Level 0) and the four geographic quadrants (Level 1) are depicted in Fig. 1, exemplifying the model's capacity to uphold logical relationships between disaggregated and aggregated projections. This furnishes a comprehensive and reconciled forecast of utilization for each of the four quadrants and the overall province of WP, extending through December 2024.

The accuracy of the Prophet model was enhanced through the inclusion of the flood variable as an exogenous regressor. As demonstrated in Table A.4, the model incorporating the categorical flood variable outperformed the one with the continuous flood variable. For districts with a historically better performance, such as Limulunga and Kalabo, the integration of flood data yielded a *good forecast* with MAPE values ranging from 10 % to 20 %. Conversely, districts with a historically weaker performance, including Sikongo and Lukulu, continued to fall within the *acceptable* or *poor forecast* categories, despite the addition of the flood regressor. This observation indicates that the influence of flood exposure on utilization is not uniform and may be confounded by unmeasured district-level factors. A visual comparison of the models' performance for these districts, contrasting the baseline Prophet model with the model that includes the categorical flood regressor, is presented in Figure A.3.

### 3.3 Interrupted time series analysis

A generalized linear mixed-effects model was employed to evaluate the influence of three major interruptions on overall utilization (Fig. 2a, Fig. 2b). The model output, which accounts for overarching temporal trends, seasonal variations, district fixed effects, and random intercepts for each health facility, is presented in Table 2. The findings confirmed a significant negative temporal trend in utilization throughout the study period (Estimate = −0.017, *p* < 0.001), alongside notable seasonal effects (*p* < 0.001). The three interruptions exhibited distinct impacts: the drought (January 2019) was associated with a significant immediate decline in utilization (Level Estimate = −0.105, *p* < 0.001), followed by a significant positive change in trend (Trend Estimate = 0.019, *p* < 0.001), indicating a period of recovery. The COVID-19 pandemic (January 2021) correlated with a significant immediate increase in utilization (Level Estimate = 0.069, *p* < 0.001), succeeded by a strong negative change in trend (Trend Estimate = −0.005, *p* < 0.001), reflecting a subsequent decrease. Finally, the complex flood vvent (i.e., February 2023 double-peak flood) demonstrated no significant immediate alteration in utilization (Level Estimate = −0.003, *p* = 0.896). Still, it was associated with a significant positive change in trend (Trend Estimate = 0.019, *p* < 0.001), suggesting a gradual recovery post-event. The time series depicting general utilization and flood depth, offering environmental context for these interruptions, is illustrated in Fig. 2a. The counterfactual analysis visually depicted the estimated impacts of each interruption. As illustrated in Fig. 2b, the observed data (points) are plotted against the model's factual prediction (solid line), which represents the average expected utilization after accounting for all variables like seasonality and underlying trends. The dashed lines represent the counterfactual scenarios. Each counterfactual shows the trajectory the model would have predicted if that interruption's specific impact (i.e., its associated level and trend changes) had not occurred. For instance, the 2019 drought was associated with a significant positive change in the recovery trend (Table 2). Consequently, the 'No Drought' counterfactual, which projects the original, less steep pre-drought trend forward, results in a substantially lower utilization estimate in subsequent years. This analysis underscores how each event, notably the drought and COVID-19, modified the underlying trajectory of health-seeking behaviour, while the model indicates that the flood event initiated a recovery trend.

**Fig. 2b:**
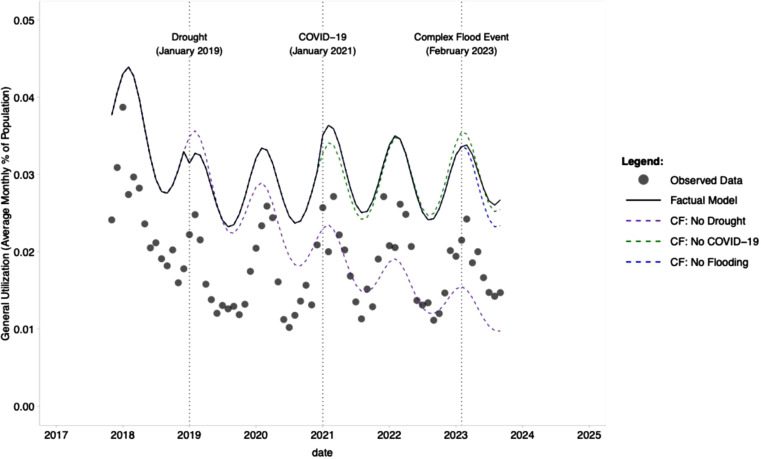
Interrupted Time Series (ITS) model of general utilization. Grey points represent the observed monthly average general utilization. The solid black line illustrates the factual prediction from the fitted model, representing the expected mean utilization. The dashed lines depict counterfactual (CF) scenarios that the model estimates would have occurred had a specific interruption's associated level and trend change not taken place. Vertical dotted lines indicate the onset of the three analyzed interruptions. This plot visualizes the immediate (level) and long-term (trend) effects of each event, with full model coefficients and 95 % confidence intervals provided in Table A.4.

**Table 2:** Interrupted time series regression results for general utilization with fixed and random effects (October 2017 to September 2023).

Predictor	Estimate	95 % CI	Std. Error	P value
(Intercept)	**–7.85882**	(–7.96585, –7.75179)	0.05461	< 0.001 ***
**Administrative District**				
*Kalabo* (*Ref*.)				
Limulunga	0.01684	(–0.11912, 0.15280)	0.06937	0.808
Lukulu	0.14297	(–0.10229, 0.38824)	0.12514	0.253
Mitete	0.02353	(–0.26861, 0.31568)	0.14906	0.875
Mongu	–0.03173	(–0.17308, 0.10962)	0.07212	0.660
Nalolo	–0.09061	(–0.26944, 0.08822)	0.09124	0.321
Sikongo	–0.04963	(–0.44929, 0.35002)	0.20391	0.808
**Time**				
Months since start	**–0.01737**	(–0.02145, –0.01329)	0.00208	< 0.001 ***
Sine (Harmonic)	**0.17663**	(0.16432, 0.18894)	0.00628	< 0.001 ***
Cosine (Harmonic)	**–0.02862**	(–0.04008, –0.01716)	0.00585	< 0.001 ***
**Interruptions (Shocks)**				
***January 2019***				
*Drought Onset*				
Level	**–0.10451**	(–0.15065, –0.05838)	0.02354	< 0.001 ***
Trend	**0.01906**	(0.01461, 0.02352)	0.00227	< 0.001 ***
***January 2021***				
*COVID–19 Pandemic (lagged)*				
Level	**0.06947**	(0.03069, 0.10824)	0.01978	< 0.001 ***
Trend	**–0.00487**	(–0.00748, –0.00225)	0.00133	< 0.001 ***
***February 2023***				
*Complex Flood Event ("double–peak")*				
Level	–0.00343	(–0.05484, 0.04798)	0.02623	0.896
Trend	**0.01957**	(0.00883, 0.03030)	0.00548	< 0.001 ***
**Random Effects**	**Variance**	**Std. Dev.**		
Intercept (Health Facility)	0.03784	0.19452		

**Note:** Results from a *generalized linear mixed–effects model* (GLMM) estimating changes in monthly general utilization as a percent of the population, based on an interrupted time series (ITS) framework with random intercepts for health facilities (*n* = 62). Fixed effects include overall time trends, seasonal patterns (sine and cosine harmonics), district fixed effects (*Kalabo* [*Ref*.]), and post–shock level and trend changes associated with three major interruptions: the 2019 drought, the 2021 COVID–19 pandemic (*lagged*), and the 2023 complex flood event (i.e., February 2023 double-peak flood). A Beta distribution with a logit link function was used as the outcome was a proportion. A diagnostic check of the model residuals indicated significant autocorrelation that could not be resolved due to model convergence failures; therefore, p-values should be interpreted with caution. Reported are model estimates, 95 % confidence intervals (Wald), standard errors (Std. Error), and p-values for fixed effects, along with variance and standard deviation (Std. Dev.) estimates for random effects. All models were fit using the *glmmTMB* package in R. **Levels of Significance:***p* < 0.05*, *p* < 0.01**, *p* < 0.001***.

## 4 Discussion

This study presents a quantitative, multi-model time-series analysis of health service utilization in Western Province (WP), Zambia. The findings provide robust evidence of the health system's dynamic responsiveness to environmental and pandemic-related shocks. Benchmarking results identified Prophet as the most accurate forecasting model, with a MAPE of 7.22 %. The Hierarchical Time Series (HTS) model confirmed a declining utilization trend and revealed distinct spatial patterns, with projections indicating a more stable trend in the southwestern quadrant compared to a steeper decline in the southeastern region (see Fig. 1). The incorporation of flood data as an exogenous variable enhanced predictive accuracy in certain districts, such as Kalabo, but not in others, such as Sikongo, thereby underscoring the spatial heterogeneity of these effects. Notably, the Interrupted Time Series (ITS) analysis demonstrated that the three major shocks exerted distinct and quantifiable impacts: a drought in 2019 was associated with a substantial immediate reduction in utilization (Level Estimate = −0.105, *p* < 001) and a subsequent positive recovery trend; the COVID-19 pandemic in 2021 resulted in a significant immediate increase in utilization followed by a negative trend change; and the complex flood event, specifically the double-peak flood in February 2023, although not producing a significant immediate impact, was linked to a significant positive trend change.

The aggregate findings from our forecasting and exogenous variable analyses furnish compelling quantitative evidence supporting the dynamic and spatially heterogeneous nature of health system resilience in vulnerable settings. The superior performance of the Prophet model, especially when compared with traditional methodologies such as ARIMA, corroborates an expanding body of scholarly literature that endorses flexible, machine learning-based models capable of managing non-linear trends and intricate seasonality typical of routine health data in low-resource environments.[9], [10] This selection of the model proved essential for generating precise forecasts within a data environment characterized by significant temporal variability. Our analysis additionally substantiated that the utility of external predictors, including flood data, in enhancing forecast accuracy varies across districts. The heterogeneous impacts of the flood regressor, which augmented predictive performance for districts such as Kalabo and Limulunga but had minimal influence on others like Sikongo and Lukulu, illustrate that the effect of macro-level shocks is highly contingent upon underlying local stressors and geographic context [30]. This finding reinforces the concept that a uniform approach to forecasting or intervention is unlikely to be effective and that predictive models must integrate the spatial heterogeneity of vulnerabilities within health systems.

Our Interrupted Time Series (ITS) analysis introduced an inferential perspective to these findings by demonstrating that distinct shocks produced fundamentally different system-level responses. The notable immediate decrease in utilization, followed by a recovery trajectory in response to the 2019 drought, indicates a shock that temporarily impeded access but from which the system was able to partially recover. This pattern markedly contrasts with findings from studies of the COVID-19 pandemic, which, in our analysis, correlated with a significant initial increase in utilization followed by a sustained decline. This observation is consistent with studies conducted in Ghana and Uganda, which reported complex, non-uniform effects of the pandemic on health service utilization [31]. Such a pattern may reflect a period of heightened health-seeking behaviour prompted by the infectious disease shock, succeeded by a *wear-out* effect or burnout in subsequent months. Conversely, the complex flood event (i.e., February 2023 double-peak flood) exhibited no significant immediate impact on utilization but was associated with a substantial positive trend change. This finding aligns with studies from Rwanda and Kenya, which utilized ITS analysis to demonstrate that certain system-strengthening interventions can induce a gradual increase in utilization over time rather than an immediate change [32]. These diverse responses to various shocks offer compelling evidence for a dynamic and multifaceted understanding of health system resilience, a fundamental component of the conceptual framework underpinning this study.

This study possesses multiple notable strengths. Its foremost contribution lies in the novel application of a multi-model approach. It integrates time-series forecasting, hierarchical analysis, and interrupted time series analysis to provide a comprehensive and dynamic evaluation of health system resilience. Using a substantial, multi-year longitudinal dataset encompassing 62 health facilities across an entire province also enhances the generalizability of our findings within this region, a recognized advantage over smaller-scale studies [19]. A further strength is our use of a unique, curated flood exposure variable derived from a hydrodynamic inundation model and utilized in peer-reviewed publications by an international research collaboration [22]. This provides a more precise and robust measure of environmental shock than is typically available in similar studies. Lastly, while the HMIS database can be affected by issues related to data quality and completeness, the data used for the primary outcome of general utilization in this study was relatively complete, with a low missingness rate of approximately 5 %. This is a notable strength, as studies have shown that data quality can significantly impact the stability and accuracy of model estimates, especially in low-resource settings [33]. Nevertheless, it is imperative to interpret our findings considering several limitations. The ecological study design precludes inferences at the individual risk level, and the observational nature of the data prevents establishing definitive causal relationships [34]. Moreover, the flood exposure variable, derived from hydrodynamic modelling and applied at the district level, may obscure finer-scale, localized variations in flood intensity and impact. Finally, a diagnostic evaluation of the residuals from the ITS model revealed significant autocorrelation that could not be rectified due to model convergence failures. This is a common challenge in time-series analysis and means that the p-values from the ITS model should be interpreted with caution [35].

The findings of this study provide a compelling justification for policy interventions aimed at mitigating the adverse synergistic interaction between environmental shocks and pre-existing health system stressors. This strategy is central to the econosyndemic framework, which our research empirically substantiates. The evidence of geographically heterogeneous responses strongly argues against a uniform approach to intervention, highlighting the imperative for decentralized and data-driven strategies [36]. For health planners, this entails prioritizing investments in districts where the detrimental interaction between elevated environmental risks and limited health system capacity is most pronounced. The predictive capabilities of our models, especially the Prophet model's capacity to deliver precise quarterly forecasts, can be harnessed to transition from reactive to proactive strategies for enhancing health system resilience [37]. For instance, these predictive models can function as an early warning system, enabling health systems to pre-position essential medical supplies, reinforce referral transport networks, and ensure sufficient staffing in high-risk areas in anticipation of events such as seasonal floods. Such a shift is vital for cultivating genuine health system resilience and protecting essential services amidst climate-related uncertainties [38].

## 5 Conclusion

This study provides innovative empirical evidence that the intricate interaction between acute environmental shocks and chronic stressors on health systems exerts a dynamic influence on health service utilization within a rural setting vulnerable to climate impacts. The employment of a multi-model time-series methodology, which integrates forecasting with Interrupted Time Series analysis, elucidates how these synergistic effects generate geographically heterogeneous patterns of disruption and resilience. Ultimately, the findings suggest the potential utility of adopting multi-model predictive strategies of implementing targeted, data-informed interventions to bolster local health system capacity, thereby contributing to the progress of Universal Health Coverage and serving as a fundamental element of climate change adaptation. Moreover, this research delineates several critical avenues for future scholarly inquiry. Longitudinal studies are vital to validate the causal pathways outlined in our models and to establish links between observed utilization trends and subsequent health outcomes. Future research would benefit significantly from integrating more granular data, such as higher-resolution flood metrics, facility-specific preparedness indicators, and household-level information on health-seeking behaviour and socioeconomic variables. Most importantly, future investigations must prioritize implementation science: the design, implementation, and rigorous evaluation of targeted, contextually tailored interventions, which our findings suggest are indispensable.

## CRediT authorship contribution statement

**Cameron B. Chiarot:** Writing – review & editing, Writing – original draft, Visualization, Validation, Software, Methodology, Investigation, Formal analysis, Data curation, Conceptualization. **Craig R. Janes:** Writing – review & editing, Funding acquisition. **Fastone Goma:** Writing – review & editing. **Karen A. Grépin:** Writing – review & editing. **Abel Torres-Espin:** Writing – review & editing. **Elizabeth J. Mroz:** Writing – review & editing, Data curation. **Mark W. Smith:** Writing – review & editing, Data curation. **Zahid A. Butt:** Writing – review & editing, Supervision.

## Declaration of competing interest

The authors declare that they have no known competing financial interests or personal relationships that could have appeared to influence the work reported in this paper.

## Appendix Supplementary materials

Image, application 1

## Footnotes

Supplementary material associated with this article can be found, in the online version, at doi:10.1016/j.joclim.2026.100672.

## Footnotes

1 The analytical framework used in this study, defined as the econosyndemic, is described in a related manuscript submitted for publication [Author et al., 2025].

2 The SCSO-PUR methodology used in this study to calculate proportion of general utilization was described in a related manuscript submitted for publication [Author et al., 2025].
