# Supplementary material for: Health system resilience: quantifying the dynamic impact of environmental shocks on health service utilization using an interrupted time series and time-series forecasting approach in Western Province, Zambia
Source: J Clim Chang Health. 2026 Apr 10;28:100672. doi: 10.1016/j.joclim.2026.100672 (PMC13092025; doi:10.1016/j.joclim.2026.100672)
Supplement: Supplementary file 1 [file mmc1.docx]

**Appendix A**

**Table A.1.** Forecast model performance comparison for general utilization.

| **Model** | **R pkg [function]** | **RMSE** | **MAE** | **MAPE (%)** | **Description** |
| --- | --- | --- | --- | --- | --- |
| ARIMA | *forecast*  [auto.arima()] | 0·000029 | 0·000025 | 16·30 | Baseline autoregressive integrated moving average |
| SARIMA | *forecast* [auto.arima()] | 0·000029 | 0·000025 | 16·30 | Seasonal autoregressive integrated moving average |
| ETS | *forecast* [ets()] | 0·000025 | 0·000022 | 13·56 | Exponential smoothing |
| Prophet | *Prophet* [prophet()] | **0·000015** | **0·000012** | **7·22** | Additive; captures trends, seasonality, and changepoints |
| **Note**: Forecast accuracy of four time series models used to predict general health service utilization in Western Province, Zambia. All models were trained on data from Oct 2017 to Dec 2022 (*n* = 63 months) and tested on Jan 2023 to Oct 2023 (*n* = 10 months). Accuracy was evaluated on total monthly utilization (i.e., outpatient visits) across all study facilities. The Prophet model demonstrated the highest forecast accuracy (MAPE = 7·22%) on the test set and was selected for further modeling. **Abbreviations: ARIMA** Autoregressive Integrated Moving Average, **SARIMA** Seasonal ARIMA, **ETS** Exponential Smoothing, **RMSE** Root Mean Squared Error, **MAE** Mean Absolute Error, **MAPE** Mean Absolute Percentage Error (lower is ideal). | | | | | |

**Figure A.1.** Prophet forecast of general utilization. The Prophet model forecast for aggregate monthly general utilization from October 2017 to September 2024. The model, which captures trend and seasonality, was trained on monthly data through December 2022 and evaluated using a 9-month test set from January to September 2023. The solid line shows the model's point forecast, while the shaded area indicates the 95% confidence interval.

**Table A.2.** Forecasted general utilization by administrative district and quarter (September 2023 to September 2024).

| **Quarter–Year /**  **Admin. District** | **District**  **Pop.** | **Baseline**  **(%)** | **Baseline Visits** | **Change from Baseline (%)** | **Forecast**  **(%)** | **95% CI** –  **Forecast %** | **Predicted Visits** | **95% CI** –  **Predicted Visits** | **Forecast**  **Utilize Flag** |
| --- | --- | --- | --- | --- | --- | --- | --- | --- | --- |
| **Q4 2023^[[1]](#footnote-1)^** |  |  |  |  |  |  |  |  |  |
| Kalabo | 48919·2 | 0·0209 | 30·3 | 7·1 | 0·0224 | (0·018–0·027) | 32·8 | (26·1–39·1) | .. Utilize |
| Limulunga | 61336·4 | 0·0124 | 22·9 | 10·3 | 0·0136 | (0·010–0·017) | 25·1 | (18·6–31·9) | .. Utilize |
| Lukulu | 9969·7 | 0·0144 | 4·7 | 35·0 | 0·0194 | (0·011–0·029) | 5·8 | (3·2–8·6) | .. Utilize |
| Mitete | 4992·5 | 0·0266 | 3·9 | –2·9 | 0·0258 | (0·017–0·035) | 3·9 | (2·6–5·2) | .. Over |
| Mongu | 80628·8 | 0·0097 | 23·0 | 33·1 | 0·0130 | (0·009–0·016) | 31·4 | (22·9–39·7) | .. Severe Under |
| Nalolo | 25326·6 | 0·0143 | 10·9 | 1·2 | 0·0145 | (0·011–0·018) | 11·0 | (8·4–13·5) | .. Under |
| Sikongo | 5139·3 | 0·0070 | 1·1 | 71·3 | 0·0120 | (0·005–0·019) | 1·8 | (0·8–2·9) | .. Severe Under |
| **Q1 2024** |  |  |  |  |  |  |  |  |  |
| Kalabo | 48919·2 | 0·0248 | 36·6 | 22·0 | 0·0303 | (0·026–0·035) | 44·4 | (37·8–50·7) | .. Over |
| Limulunga | 61336·4 | 0·0194 | 33·9 | 24·2 | 0·0241 | (0·020–0·028) | 44·3 | (37·4–51·3) | .. Over |
| Lukulu | 9969·7 | 0·0341 | 11·7 | –9·4 | 0·0309 | (0·022–0·040) | 9·2 | (6·5–11·9) | .. Over |
| Mitete | 4992·5 | 0·0272 | 4·1 | 2·2 | 0·0278 | (0·019–0·037) | 4·2 | (2·9–5·5) | .. Over |
| Mongu | 80628·8 | 0·0149 | 37·1 | 29·9 | 0·0194 | (0·016–0·023) | 46·9 | (38·2–55·4) | .. Utilize |
| Nalolo | 25326·6 | 0·0189 | 14·7 | 16·1 | 0·0219 | (0·019–0·025) | 16·6 | (14·1–19·1) | .. Utilize |
| Sikongo | 5139·3 | 0·0200 | 3·1 | –15·9 | 0·0168 | (0·010–0·024) | 2·6 | (1·6–3·7) | .. Utilize |
| **Q2 2024** |  |  |  |  |  |  |  |  |  |
| Kalabo | 48919·2 | 0·0206 | 29·4 | 16·4 | 0·0240 | (0·019–0·028) | 35·2 | (28·5–41·8) | .. Over |
| Limulunga | 61336·4 | 0·0193 | 36·4 | –1·1 | 0·0191 | (0·016–0·023) | 35·2 | (28·6–42·0) | .. Utilize |
| Lukulu | 9969·7 | 0·0174 | 5·1 | 40·9 | 0·0245 | (0·016–0·033) | 7·3 | (4·7–10·0) | .. Over |
| Mitete | 4992·5 | 0·0172 | 2·6 | 36·7 | 0·0236 | (0·015–0·032) | 3·5 | (2·3–4·8) | .. Over |
| Mongu | 80628·8 | 0·0131 | 32·6 | 12·0 | 0·0147 | (0·011–0·018) | 35·6 | (27·0–44·6) | .. Under |
| Nalolo | 25326·6 | 0·0154 | 12·0 | 0·6 | 0·0155 | (0·012–0·019) | 11·8 | (9·3–14·3) | .. Under |
| Sikongo | 5139·3 | 0·0187 | 2·9 | 3·4 | 0·0193 | (0·012–0·026) | 3·0 | (1·9–4·0) | .. Utilize |
| **Q3 2024** |  |  |  |  |  |  |  |  |  |
| Kalabo | 48919·2 | 0·0157 | 23·9 | 16·9 | 0·0184 | (0·014–0·023) | 26·9 | (20·2–33·4) | .. Utilize |
| Limulunga | 61336·4 | 0·0122 | 23·0 | –14·7 | 0·0104 | (0·007–0·014) | 19·2 | (12·4–26·1) | .. Severe Under |
| Lukulu | 9969·7 | 0·0161 | 3·7 | –34·8 | 0·0105 | (0·002–0·019) | 3·1 | (0·5–5·8) | .. Severe Under |
| Mitete | 4992·5 | 0·0150 | 2·3 | 52·6 | 0·0230 | (0·014–0·031) | 3·4 | (2·1–4·7) | .. Utilize |
| Mongu | 80628·8 | 0·0125 | 29·2 | –12·9 | 0·0108 | (0·007–0·014) | 26·2 | (17·7–34·9) | .. Severe Under |
| Nalolo | 25326·6 | 0·0109 | 7·8 | 14·8 | 0·0125 | (0·009–0·016) | 9·5 | (7·0–12·0) | .. Severe Under |
| Sikongo | 5139·3 | 0·0157 | 2·4 | –32·7 | 0·0105 | (0·004–0·018) | 1·6 | (0·6–2·7) | .. Severe Under |
| **Note*:*** Forecasted general utilization by administrative district and quarter (Oct 2023 – Sep 2024). Flags represent forecasted utilization levels based on percentage thresholds: .. **Severe Under–utilization** < 0·013%, .. **Under–utilization** = 0·013–0·018%, .. **Utilization** = 0·018–0·023%, .. **Over–utilization** > 0·023%. Forecasts were compared to baseline values from the equivalent quarters in 2022–2023. The forecast utilization flag thresholds presented in Table 3a were defined based on the quartiles of the historical distribution of monthly general utilization from 2017-2023 Refer to table 4 for equations. | | | | | | | | | |

**Table A.3.** Summary of forecast and translation metrics used in Table 3.

| **Model Metric** | **Equation and Description** |
| --- | --- |
| Baseline Visits | $Baseline \% \times\left( Population\times3 months \right)$ |
| Forecast % | $\hat{y}_{t}=Prophet prediction at time t$ |
| Forecasted Visits | $Forecast \% \times\left( Population\times3 months \right)$ |
| 95% CI – Forecast % (Range) | $\hat{y}_{t}\in\left( \hat{y}_{t}^{\mathrm{lower}},\hat{y}_{t}^{\mathrm{upper}} \right)\times Population\times3$ |
| 95% CI – Forecasted Visits (Range) | $CI \% bounds\times\left( Population\times3 months \right)$ |
| Change from Baseline (%) | $\left( \frac{Forecast \%-Baseline \%}{Baseline \%} \right)\times100$ |
| Forecast Utilization Flag Thresholds | Severe Under (<0·013), Under (0·013–0·018), Utilize (0·018–0·023), Over (>0·023) |
| **Note:** $\hat{y}_{t}$ represents the predicted proportion of general utilization (i.e., visits) at time $t$ from the Prophet model. Confidence Intervals (CI) reflect model–based uncertainty in the forecasts. | |

**Figure A.2.** District-level forecasts of general utilization from seven individual Prophet models. Panel A shows the model validation, comparing the forecast (dashed blue line) against the actual observed data (green points) on the 9-month hold-out test set from January to September 2023. Panel B displays the full 12-month future forecast, projecting utilization trends from November 2023 to October 2024.

**Table A.4.** Forecast accuracy of flood–augmented Prophet models by administrative district.

| **Model Type / Administrative District** | **RMSE** | **MAE** | **MAPE (%)** | **Interpretation** |
| --- | --- | --- | --- | --- |
| **Categorical Flood Model** |  |  |  |  |
| Kalabo | 0·0000378 | 0·0000290 | **14·1** | .. Good forecast |
| Limulunga | 0·0000295 | 0·0000250 | **13·4** | .. Good forecast |
| Lukulu | 0·0000733 | 0·0000566 | **27·4** | .. Acceptable |
| Mitete | 0·0000556 | 0·0000428 | 25·6 | .. Acceptable |
| Mongu | 0·0000343 | 0·0000285 | 18·0 | .. Good forecast |
| Nalolo | 0·0000254 | 0·0000207 | 14·2 | .. Good forecast |
| Sikongo | 0·0000929 | 0·0000777 | **N/A** | .. Poor forecast |
| **Continuous Flood Model** |  |  |  |  |
| Kalabo | 0·0000448 | 0·0000363 | 18·0 | .. Good forecast |
| Limulunga | 0·0000307 | 0·0000258 | 13·8 | .. Good forecast |
| Lukulu | 0·0000753 | 0·0000594 | 29·9 | .. Acceptable |
| Mitete | 0·0000493 | 0·0000384 | 22·8 | .. Acceptable |
| Mongu | 0·0000312 | 0·0000256 | 16·3 | .. Good forecast |
| Nalolo | 0·0000278 | 0·0000225 | 15·8 | .. Good forecast |
| Sikongo | 0·0000876 | 0·0000714 | N/A | .. Poor forecast |
| **Note:** Performance of Prophet models augmented with flood level as an exogenous predictor variable (i.e., Flood level as categorical [low, medium, and high] and flood depth [m] as continuous. RMSE, MAE, and MAPE (%) are shown by district. Forecasts were considered “good” when MAPE < 20%, and “acceptable” up to 50%. **Abbreviations:** **RMSE** Root Mean Squared Error, **MAE** Mean Absolute Error, **MAPE** Mean Absolute Percentage Error. ***MAPE Range (%) Interpretation:*** .. < 10 Excellent forecast, .. 10–20 Good forecast, .. 20–50 Acceptable, .. > 50 Poor forecast. **Bold Text MAPE %:** used in Figure 4. MAPE is not applicable (N/A) for Sikongo due to zero values in the test set; model performance for this district was assessed using MAE. | | | | |

**Figure A.3.** Forecast accuracy with flood regressor. Comparison of Prophet model forecast accuracy with and without the flood category as an exogenous regressor. The figure highlights two districts with good forecast performance (Limulunga, Kalabo) and two with weaker performance (Sikongo, Lukulu) to demonstrate model variability, as measured by Mean Absolute Percentage Error (MAPE) and Mean Absolute Error (MAE). The vertical dotted line indicates the transition from the training phase to the test phase.

1. Observed utilization for Q4 2023, based on available data from October 2023, was as follows: Kalabo (0·017%), Limulunga (0·013%), Lukulu (0·015%), Mitete (0·020%), Mongu (0·014%), Nalolo (0·013%), and Sikongo (0·017%). [↑](#footnote-ref-1)
